# High impact of molecular surveillance on hepatitis A outbreak case detection in Sweden: a retrospective study, 2009 to 2018

**DOI:** 10.2807/1560-7917.ES.2021.26.9.1900763

**Published:** 2021-03-04

**Authors:** Maximilian Riess, Theresa Enkirch, Lena Sundqvist, Josefine Lundberg Ederth

**Affiliations:** 1 Public Health Agency of Sweden, Department of Microbiology, Solna, Sweden; 2 European Public Health Microbiology Training Programme (EUPHEM), European Centre for Disease Prevention and Control (ECDC), Solna, Sweden; 3 Public Health Agency of Sweden, Department of Communicable Disease Control and Health Protection, Solna, Sweden; 4 Public Health Agency of Sweden, Department of Public Health Analysis and Data Management, Solna, Sweden

**Keywords:** hepatitis A, hepatitis A virus, HAV, sequence-based typing, sequence analysis, surveillance, outbreak

## Abstract

**Background:**

Swedish hepatitis A surveillance includes sequence-based typing, but its contribution to outbreak detection in relation to epidemiological investigations has not been fully evaluated.

**Aim:**

To evaluate the role of sequence-based typing in hepatitis A outbreak detection and to describe the hepatitis A epidemiology in Sweden to improve surveillance.

**Methods:**

We retrospectively investigated hepatitis A virus sequences of 447 cases notified in Sweden 2009–18. We performed a phylogenetic analysis of evolutionary distances to identify cases with similar virus sequences (≥ 459/460 identical nt in the VP1/P2A junction). Unique sequences, dyads and sequence-based clusters (SBCs) were identified. We linked non-sequenced cases by epidemiological information and retrospectively assessed the value of typing for outbreak identification.

**Results:**

Fifty-five percent (n = 542/990) of the notified hepatitis A cases were referred to the Public Health Agency of Sweden for typing and 447 (45%) were sequenced successfully. Subgenotypes included IA (42.5%, n = 190), IB (42.7%, n = 191) and IIIA (14.8%, n = 66). Phylogenetic analysis identified 154 unique sequences, 33 dyads (66 cases) and 34 SBCs (227 cases). The combination of molecular and epidemiological data revealed 23 potential outbreaks comprising 201 cases. Cases were linked by sequence (59%, n = 118), epidemiological data (11%, n = 23) or both (30%, n = 60). Typing was needed to identify 15 of 23 potential outbreak signals.

**Conclusion:**

Sequence-based typing contributed substantially to detecting clustering cases and identifying outbreaks in Sweden. The results show routine sequence-based typing detects outbreaks, promotes timely outbreak investigations and facilitates international collaboration.

## Introduction

Hepatitis A virus (HAV) infections have gained increased awareness in Europe in recent years. This is mainly due to large and prolonged European-wide outbreaks [1-10] and an interrupted trend of decreasing hepatitis A notification rates [11]. In Sweden, the number of yearly reported hepatitis A cases is low [11], but outbreaks occur at the local/regional and national levels and Swedish cases are repeatedly identified as part of European outbreaks.

HAV is primarily transmitted through the faecal/oral route and sources of transmission include contaminated water or food products, close contacts (e.g. at school, shared household or intimate contacts), contaminated blood products and shared equipment for intravenous drug use. The incubation period is ca 28 days (range: 15–50 days) [12,13]. Six HAV genotypes are known, of which I-III are infectious for humans, and can be further divided in subgenotypes A and B, respectively [5,12]. Genotype I is most prevalent globally, genotype III appears predominantly in south Asia and Eastern Europe and genotype II is rarely reported [14]. The mutation rate of the HAV genome is slow [15]. During hepatitis A outbreak investigations clusters are commonly defined from sequences that are identical or have small genetic variations. In international and prolonged outbreak situations, acquisition of larger variations of up to 3 nucleotides (nt) difference in a ca 460 nt fragment (0.7%) have been reported [1,4,7,16].

It is essential to rapidly and comprehensively identify outbreak cases in order to understand the extent and likely source of the outbreak. Sequence-based typing is critical for connecting cases and determining the source of the outbreak, especially for cross-border outbreaks [1,4-7,9,10,17,18]. With sequence-based typing, a HAV strain of a contaminated food item may be linked to the outbreak strain and identified as the source [1]. Yet, as per the last systematic assessment done in 2016, ca 50% of European countries do not perform sequence-based typing, in particular those countries with high endemicity [19]. Therefore, the number of domestic outbreaks and cases linked to European outbreaks are likely underestimated.

In Sweden, hepatitis A is a notifiable disease by law and is monitored by a national passive surveillance system. Since 2006, sequence-based typing of clinical samples has been carried out by the Public Health Agency of Sweden (PHAS) as part of the national microbiological surveillance programme. Changes in the programme’s focus on domestic versus travel-associated cases has led to varying proportions of samples being referred for sequencing. Obtained sequences are stored and continuously analysed.

This study aimed to retrospectively describe the epidemiology of hepatitis A in Sweden from 2009 to 2018 and investigate the role of HAV sequence-based typing on outbreak identification during this period. Furthermore, the value of sequence-based typing is discussed and suggestions for improved HAV molecular surveillance are presented.

## Methods

### Study design and data source

All confirmed hepatitis A cases from 2009–18 mandatorily notified in the Swedish electronic system for communicable diseases surveillance (SmiNet), were included in the study. SmiNet is a common platform shared between the regional and national levels of public health agencies in Sweden.

**Figure 1:**
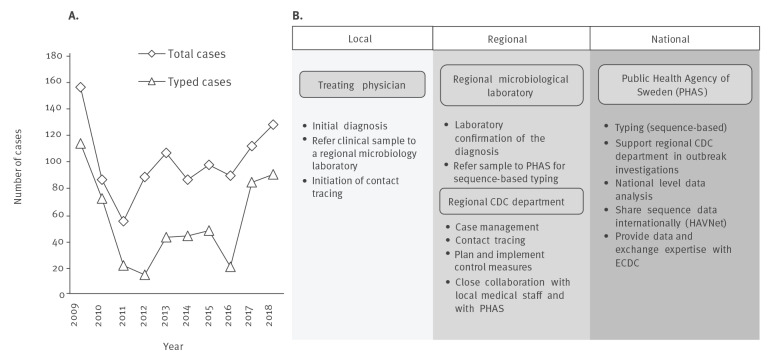
(A) Number of notified hepatitis A cases, Sweden, 2009–2018 and (B) key public health actors involved in the Swedish national hepatitis A surveillance programme

For a case to be confirmed, a blood sample from a patient with clinically suspected hepatitis A is referred to a regional microbiological laboratory for anti-HAV IgM and/or HAV RNA detection. If positive, the treating physician and the laboratory notify the confirmed case to SmiNet. The regional County Council Departments of Communicable Disease Control and Prevention (CDC departments) work largely autonomously, they carry out case management and contact tracing, evaluate data at regional level and implement control measures. PHAS evaluates the SmiNet surveillance data at national level, has a coordinating role in national outbreak investigations and provides the sequence-based typing service (Sanger sequencing). This is free of charge and PHAS reports the results in SmiNet, visible to the CDC departments. For sequence-based typing, the regional microbiological laboratories refer samples to PHAS as part of an agreement between the respective regional CDC departments and PHAS, depending on the focus of the molecular surveillance program on domestic and travel-associated HAV infections (Figure 1). The genomic HAV region sequenced at PHAS covers the 460 nt VP1/P2A-region recommended for typing by HAVNet [14,20], a global network of HAV laboratories that provides a common protocol for typing, and hosts a database for online sharing of HAV sequences and metadata. At PHAS, new sequences are compared with those encountered previously, anonymised sequence data is shared internationally through HAVNet and other international networks. This includes collaborations facilitated through the European Centre for Disease Prevention and Control (ECDC) such as the Epidemic Intelligence Information System for Food- and Waterborne Diseases and Zoonoses (EPIS-FWD). EPIS-FWD allows the sharing of HAV sequences of ongoing outbreaks along with epidemiological descriptions and enables communication between public health professionals in the network.

CDC department: Regional County Council Departments of Communicable Disease Control and Prevention; ECDC: European Centre for Disease Prevention and Control; HAV: hepatitis A virus; PHAS: Public Health Agency of Sweden.

For each case, data obtained from SmiNet included sex, age, travel status (suspected country of infection), date of symptom onset, sampling date and information regarding the HAV infection (suspected source of infection, any established epidemiological link to another case, symptomatic/asymptomatic status). Additional epidemiological information (links to other cases, details on symptoms and suspected route of transmission) communicated between PHAS and the CDC departments during the study period was also included in the evaluation. Swedish population data were obtained from the national statistics office, Statistics Sweden [21].

### Definitions

#### Swedish case definition

Sweden applies the European case definition for hepatitis A [22]. A possible hepatitis A case is defined as having a clinical picture consistent with a hepatitis A infection and with an epidemiological link to another case or source. Confirmation of a case requires detection of a HAV-specific antibody reaction indicating an acute infection (IgM) and/or the detection of HAV RNA.

#### Definition of sequence-based clusters and outbreaks

Sequences were analysed for similarity of ≥ 99.7% (≥ 459nt/460nt) and identified as (i) HAV sequences of sporadic cases if no other similar sequence was found; (ii) dyads if two similar sequences were found; (iii) sequence-based clusters (SBCs) if three or more similar sequences or one or more sequences matching a European outbreak case definition were found. We added further cases to the SBCs if they had an epidemiological link to a SBC case. An epidemiological link was considered if a case had contact with a case in a cluster (e.g. lived in the same household, visited the same school or work place or leisure activities or was a close or intimate contact) or had been exposed to the same suspected or confirmed vehicle of transmission.

A potential outbreak was considered if one or more cases in the SBC matched to a European outbreak strain, or if three or more cases in a SBC were infected less than 8 weeks apart (twice the mean incubation time) and/or shared an epidemiological link. Cases in the same household or family were counted as one case in the assessment of outbreaks. Investigations of European HAV outbreaks have been coordinated by the ECDC and updates were published in rapid risk assessments (RRA).

We evaluated the contribution of sequence-based typing to outbreak identification by defining each outbreak case as linked by (i) sequence only; (ii) epidemiological information only; (iii) both types of information. We investigated how often sequence information contributed to cluster sizes meeting or exceeding our predefined outbreak criteria of three cases and international outbreak threshold of one case linked to an international outbreak strain in the European Union/European Economic Area (EU/EEA).

### Data analysis

HAV sequences were aligned using CLC Main Workbench version 7.9 (Qiagen, Aarhus, Denmark). MEGA7 software version 7.0.14 was used to compute a phylogenetic tree from this alignment based on the 460nt HAVNet region using the neighbour-joining method [23]. Bootstrap analysis was computed with 1,000 replicates. Evolutionary distances were computed using the Tamura-Nei method [24]. Data management and statistical analysis were performed using STATA version 15.0 (StataCorp, Texas, United States).

Categorical variables were described as proportions and 95% confidence intervals (CI), and continuous variables were described using means and standard deviations (SD), or medians, ranges or interquartile ranges (IQR). Variables were compared using chi-squared tests for categorical variables, and t-tests or the non-parametric Wilcoxon rank-sum test for continuous variables. Trends were assessed using an extension of the Wilcoxon rank-sum test. Observations with missing values for variables under comparison were excluded from the respective analyses. Time to sampling was calculated as the time between the date of symptom onset and date of sampling. We used an alpha level of 0.05 for all statistical tests. Stata outputs of p < 0.000 were reported as p < 0.001.

### Ethical statement

This study only included anonymised surveillance data without personal identifiers, therefore no ethical approval was needed according to Swedish national regulations. This study did not affect the diagnosis or the therapeutic strategy.

## Results

### Study population and hepatitis A epidemiology characteristics

During the 10-year study period, 990 confirmed hepatitis A cases were notified to SmiNet with a median of 92 reported cases per year (range: 54–154) (Figure 1A). This reflects a median notification rate of 0.9 cases per 100,000 citizens and year (range: 0.6–1.7). Approximately half the study population was male (52%, n = 518), and 52% were travel-associated (n = 513) (Table 1). The median age of cases was 17 years (range: 0–100; IQR: 7–37) and 46% (n = 453) of the cases belonged to the two youngest age groups (0–4 year olds and 5–14 year olds) (Table 1). Among the 990 confirmed cases, 14% (n = 135) were asymptomatic and identified through contact tracing of a confirmed case, or in rare cases by screenings of asylum seekers. Within the different age groups, the proportion of asymptomatic cases was highest among children 0–4 years old (34%, n = 54/161) followed by children 5–14 years old (16%, n = 47/292). This percentage strongly decreased with increasing age to 4–8% in all other age groups (3–14 asymptomatic cases) (trend test, p < 0.001) (data not shown).

**Table 1:** Characteristics of notified hepatitis A cases, Sweden, 2009–2018 (n = 990)

Case characteristics	Median	IQR	Range
Number of cases notified, n/year	92	86–103	54–154
Notifications per 100,000 population	0.9	0.9–1.1	0.6–1.7
Age (years)	17	7–37	0–100
**Case characteristics**	**n**	**%**	
All cases	990	100	
**Sex**			
Female	471	47.6	
Male	518	52.4	
Missing	1	0.1	
**Travel status**			
Domestic	376	38.0	
Travel-associated	513	51.8	
Missing	101	10.2	
**Age group (years)**			
0–4	161	16.3	
5–14	292	29.5	
15–24	169	17.1	
25–44	197	19.9	
45–64	112	11.3	
≥ 65	59	5.9	
**HAV genotypes identified^a^**			
IA	190	42.5	
IB	191	42.7	
IIIA	66	14.8	

HAV: hepatitis A virus; IQR: interquartile range.

^a^ No cases of genotypes II and IIIB were detected.

Data source: Swedish hepatitis A surveillance.

### Sequence-based typing of confirmed hepatitis A cases

**Figure 2:**
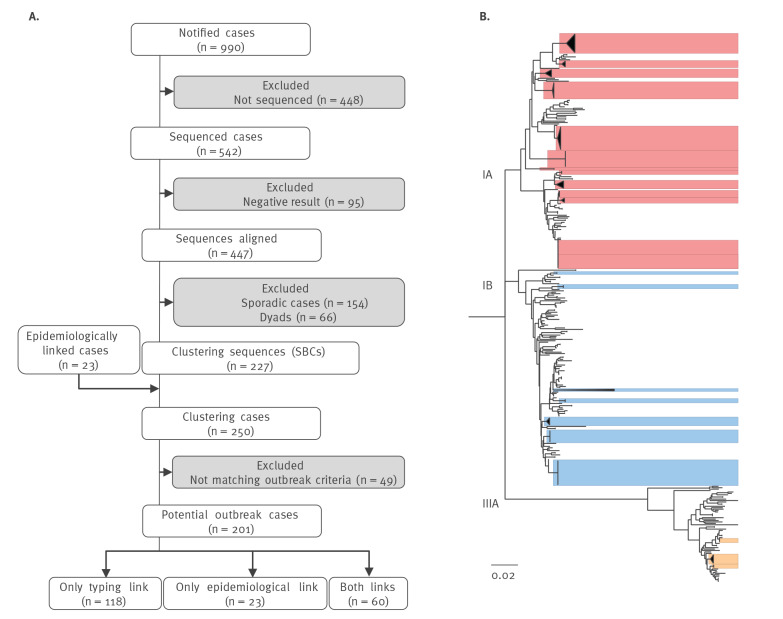
(A) Flowchart of the identification of potential hepatitis A virus (HAV) outbreaks based on sequencing data and integrated epidemiological data and (B) phylogenetic analysis of sequences from HAV strains, Sweden, 2009–2018 (n = 447)

Of the 990 notified hepatitis A cases, samples from 542 cases (55%) were referred to PHAS for typing and 447 (45%) were successfully sequenced (Figure 2A). The majority of identified subgenotypes were IA (42.5%; n = 190 cases) and IB (42.7%; n = 191 cases), with a minority of cases infected with subgenotype IIIA (14.8%; n = 66 cases) (Table 1). The frequency of referral for typing was different among age groups and increased with age (trend test, p < 0.001) (Table 2). No difference in the number of male or female cases being referred for typing (p = 0.911) was observed. Symptomatic cases were more likely to be referred for sequencing than asymptomatic cases (p < 0.001), also, domestic cases were more likely to be referred than travel-associated cases (p < 0.001) (Table 2). We also assessed the time between date of symptom onset and date of sampling and found a median of 7 days (IQR: 5–12; n = 318). However, samples with positive sequencing results (7 days; IQR: 5–11; n = 299) had a significantly shorter time between symptom onset and sampling than samples where no sequence could be obtained (17 days; IQR: 8–23; n = 19) (p < 0.001) (data not shown).

SBC: sequence-based cluster.

Branching points of genotypes IA, IB and IIIA are indicated by their label. Cases belonging to independent clusters are highlighted and size of the blocks corresponds to the number of sequenced cases in the potential outbreak. Colour coding refers to genotypes: IA (red), IB (blue), IIIA (orange). The scale bar indicates the number of nucleotide substitutions per site (0.02).

**Table 2:** Referral for hepatitis A virus sequence-based typing among cases by sex, age, travel status and being asymptomatic, Sweden, 2009–2018 (n = 990)

Case characteristics	Sequenced		Not sequenced		Chi-squared test
	n	%	n	%	p value
Total	542	54.8	448	45.2	–
**Sex**					
Female	259	55.0	212	45.0	p = 0.911
Male	283	54.6	235	45.4	
Missing	0	0.0	1	100.0	Not included
**Age group (years)**					
0–4	51	31.7	110	68.3	p < 0.001
5–14	122	41.8	170	58.2	
15–24	96	56.8	73	43.2	
25–44	145	73.6	52	26.4	
45–64	86	76.8	26	23.2	
≥ 65	42	71.2	17	28.8	
**Travel-associated**					
No	256	68.1	120	31.9	p < 0.001
Yes	252	49.1	261	50.9	
Missing	34	33.7	67	66.3	Not included
**Asymptomatic**					
No	451	59.0	314	41.0	p < 0.001
Yes	50	37.0	85	63.0	
Missing	41	45.6	49	54.4	Not included

Data source: Swedish hepatitis A surveillance.

### Outbreak identification and contribution of sequence-based typing

A phylogenetic tree was computed, and all obtained 447 sequences (Figure 2B) were analysed for similarity. We identified 154 sequences as belonging to sporadic cases (34%), 66 sequences clustered in 33 dyads (15%) and 227 sequences clustered in 34 SBCs (51%). The SBCs varied in size and contained up to 28 sequences. The proportion of sequences from travel-associated cases within these three categories were 65% (100/154), 45% (30/66) and 32% (73/227) respectively, and the categories had a similar age group distribution (p < 0.537).

An additional 23 non-sequenced cases could be linked to SBCs by epidemiological information, which led to a total of 250 clustering cases. Forty-nine cases were discarded as they neither occurred within 8 weeks of another case in the cluster, nor did they share a known epidemiological link to another case in the cluster. The remaining 201 potential outbreak cases were defined as 23 independent potential outbreaks (Figure 2B). These were spread across the whole study period (Table 3) and the majority were of subgenotype IA (n = 13; 57%) followed by subgenotypes IB (n = 7; 30%) and IIIA (n = 3; 13%) (Table 3). Sweden is divided into 21 counties and potential outbreak cases were distributed over on average three counties (SD ± 2 counties; range: 1–8). Moreover, for 13 of the 23 national outbreaks, we found strains that were detected elsewhere in Europe at the time (Table 3).

**Table 3:** Characteristics of potential hepatitis A virus outbreaks and outbreak cases, Sweden, 2009–2018 (n = 201)

Outbreak number	Year	Subgenotype	Ref	Ref strain^a^	N	Identification of cases by			Detected elsewhere in EU/EEA at the time	Typing essential for identification
						n (typing)	n (epi)	n (both)		
1	2009	IA	NA	VRD_173_2018	4	4	0	0	Yes	Yes
2	2009	IA	NA	51210	10	3	2	5	No	No
3	2009–2010	IA	NA	48535	18	8	4	6	Yes	No
4	2009–2011	IA	NA	33217	28	28	0	0	Yes	Yes
5	2010	IA	NA	51210	3	3	0	0	No	Yes
6	2010	IB	NA	33057	7	7	0	0	No	Yes
7	2012	IA	[8]	34452	1	1	0	0	Yes	Yes
8	2012–2013	IB	[2,3]	KC876797	14	1	2	11	Yes	No
9	2013	IA	NA	51211	4	3	0	1	No	Yes
10	2013	IB	[2,3]	KC876799	6	2	0	4	Yes	Yes
11	2013–2014	IA	[5-7]	KF182323	11	4	1	6	Yes	No
12	2014	IA	NA	48535	13	5	3	5	No	No
13	2015	IIIA	NA	DK2018_227	3	3	0	0	No	Yes
14	2015	IIIA	NA	34996	10	2	3	5	No	No
15	2015–2016	IB	NA	35255	7	3	4	0	No	Yes
16	2016–2018	IA	[4]	VRD_521_2016	14	12	0	2	Yes	Yes
17	2017	IIIA	NA	35607	3	3	0	0	No	Yes
18	2017–2018	IA	[4]	V16–25801	4	4	0	0	Yes	Yes
19	2017–2018	IA	[4]	RIVM-HAV16–090	15	13	0	2	Yes	Yes
20	2018	IA	[10]	DK2018_231	2	1	1	0	Yes	Yes
21	2018	IB	[10]	V18–16428	2	1	1	0	Yes	Yes
22	2018	IB	NA	48629	3	0	0	3	No	No
23	2018	IB	[1]	MH730560	19	7	2	10	Yes	No
**Total**		**23**	**NA**	**NA**	**201**	**118**	**23**	**60**	**NA**	**NA**

EU/EEA: European Union and European Economic Area; N: Total number of cases; NA: not applicable; n (typing): cases linked only by typing information; n (epi): cases linked only by epidemiological information; n (both): cases linked by typing and epidemiological information; Ref: reference.

^a^ Reference strain from HAVNet, Genebank or literature.

All cases in household clusters are listed. These were counted as one case in the outbreak assessment if typing was necessary to identify the respective potential outbreak cluster.

Data source: Swedish hepatitis A surveillance.

Strains of eight SBCs reappeared an additional 1–5 times up to 6.5 years apart without causing another potential outbreak cluster. Only one HAV IA strain appeared on three occasions in the 10-year period and generated potential outbreaks in two of the occasions 4 years apart (outbreak numbers 3 and 12 in Table 3).

Each potential outbreak case was evaluated with respect to the type of information needed to link it to that potential outbreak. Cases were linked by typing information only (n = 118; 59%), epidemiological information only (n = 23; 11%) or both types of information (n = 60; 30%) (Figure 2A, Table 3).

For 15 of the 23 potential outbreaks, the predefined outbreak criteria would not have been met had typing information not been available. Therefore, typing was considered essential for the identification of these potential outbreak clusters (Table 3).

## Discussion

We observed a median yearly notification rate for HAV infections of 0.9 per 100,000 inhabitants during the 10-year study period in Sweden, comparable to other European countries [25,26]. Vaccination confers immunity against HAV infection but is not implemented free of charge in the childhood vaccination programmes of many European countries, including Sweden [25,26]. In the absence of vaccine or infection derived immunity, population susceptibility is presumably high and outbreaks are more likely to occur [25,26], as was observed by previous years’ prolonged outbreaks in Sweden and Europe [1-10]. Swedish hepatitis A surveillance and typing data were sporadically published as part of European RRAs coordinated by the ECDC or through joint European outbreak reports [1-7,10], and sequences were also shared in HAVNet. In the present study, all data from the Swedish national molecular hepatitis A surveillance from 2009–18 were compiled and analysed for the first time.

In our combined approach of performing a phylogenetic analysis of sequence similarity, integrating epidemiological data and applying outbreak detection criteria, we identified 23 potential outbreaks. Twenty-two of these had also been investigated at the time the outbreak occurred and 10 were described in peer-reviewed literature (Table 3) [1-8,10]. One outbreak was not recognised at the time even though a similar strain was detected in another European country at the same time, as this was only communicated several years later (outbreak number 7 in Table 3). This suggests that a sequence-based approach is successful at identifying outbreak signals. The published outbreak strains were linked to food vehicles [1-3,5-8], sexual transmission (men who have sex with men (MSM)) [4] and travelling to a HAV endemic country [10]. During the study period the majority of HAV infections were of subgenotype IA or IB (42.5% and 42.7%, respectively), which fits well with the generally detected subgenotypes in Europe [14].

One IA strain was detected in Sweden three times and caused known outbreaks on two occasions (outbreak numbers 3 and 12 in Table 3). This strain was likely reintroduced to Sweden on each occasion since it was circulating in central Europe during the same time. We did not detect any strain with sustained endemic circulation. Only eight strains were detected occasionally in sporadic cases, with long time spans in between. The majority of clustering cases (59%) required typing to link them to a potential outbreak cluster. Moreover, we found that two of three potential outbreak clusters required typing to be identified. This clearly underlines the relevance of HAV typing to detect outbreak cases. Typing offers a fast and unambiguous conclusion on relatedness of cases’ strains, which is especially important considering the long incubation time of hepatitis A of up to one month. This long delay from exposure to case notification makes it difficult to draw immediate conclusions on relatedness of cases by patient interviews or epidemiological information only. Typing is a useful tool for vigilant surveillance to detect outbreaks early before they become apparent by the sheer number of cases. Moreover, it is essential to detect clusters of cases that are too small to raise suspicion by case count only, as these may slowly spread over time and between countries in Europe. Typing is also useful for distinguishing circulating strains present at the same time, allowing unrelated cases to be excluded from an epidemiological investigation. This was seen several times during the study period in Sweden. In particular, in 2014 when an independent transmission of a HAV strain was recognised during a HAV outbreak in the same municipality. During 2012–14, typing was necessary to determine the different strains during multi-country outbreaks linked to frozen berries (outbreak numbers 8, 10 and 11 in Table 3) even though epidemiological information was sufficient in two of the three outbreaks to trigger an outbreak signal.

National outbreaks in Sweden were found to be distributed between counties and over time. Similarly, international outbreaks affecting several European countries were also connected to cases detected in Sweden. Wide geographical and temporal distribution of cases is usually linked to a widely distributed food source with a long shelf-life, or to travelling [19,27]. Specifically, contaminated frozen berries [1-3,5-7] and circulation of strains among MSM [4] have been implicated in European HAV outbreaks in recent years. While local outbreaks come to the attention of the regional CDC department, mostly as increased case counts, and response measures are implemented quickly, a widespread outbreak will still benefit from linking cases by sequencing. Often, case linking will trigger an investigation. In Sweden, PHAS has a national responsibility for overseeing all HAV cases, and is therefore able to assist with coordinating between regions; a collaboration that is important to link cases and identify outbreaks across regional boarders. Likewise, on a European level, HAVNet and European agencies (the ECDC’s EPIS-FWD network and the European Food Safety Authority (EFSA)) facilitate collaborations among European public health agencies and the food sector, and the sharing of sequence data. Such cooperation has proven key to quickly identifying the extent of the geographical spread of outbreaks in Europe, identifying outbreak sources and introducing targeted control measures [1,17]. These measures are necessary to prevent the spread of the pathogen in the population, especially when facing increasing population mobility and a shared European/global food market [17,28].

Overall, 55% of all samples were referred for typing. Reasons for incomplete typing were, in part, due to a previous typing policy at PHAS, which mainly focused on domestic cases. Under this policy it was not deemed necessary to type samples from all cases involved in HAV transmissions in local community settings with epidemiologically well-linked clusters, for example in a day-care centre or in a family. Moreover, according to a previous outbreak investigation [29], not all cases are sampled in these settings which likely explains the low proportion of asymptomatic cases in the 0-4 years age group found in this study (34%) compared to other reports [12]. Increased testing and typing in outbreak settings among children may be advisable in order to detect more cases and provide comprehensive typing information. Increased typing would contribute to improved outbreak detection and facilitate the implementation of preventive measures. From 2020, PHAS aims to type all notified hepatitis A cases in Sweden. This target has not yet been reached; a typing fraction of 66.7% was achieved (38/57) in 2020. 

Ninety-five samples could not be typed successfully during the study period. These samples were associated with a longer time delay between symptom onset and sampling, which likely affected the amount of viral RNA in the samples. Within 1–2 weeks after infection, viraemia occurs and may last for several more weeks, albeit at quickly declining levels [15]. Therefore, earlier sampling may lead to a further increase in positive typing frequency. Sampling of stools, where virus shedding is observed for a longer time period, could be an alternative.

To our knowledge, this is the first comprehensive evaluation of the contribution of sequence-based typing to hepatitis A outbreak identification in Sweden. Study limitations include a possible underestimation of the number of outbreak cases due to a large number of cases that were not sequenced, as well as varied completeness of the epidemiological information available for each case. It was not in the scope of this study to improve or collect those data retrospectively. We do not expect a selection bias of samples based on budget considerations since the costs for typing lay with PHAS. For a number of potential and known outbreaks, the source was unknown or suspected but not confirmed, which prevented us from analysing the impact of typing on identifying outbreak sources. Our thresholds for number of cases needed to identify potential outbreak clusters remained low and are therefore sensitive. In particular, the threshold of one case with a matching strain to an international EU/EEA outbreak cluster is very low, but was necessary to detect single cases of cross-border events, which are otherwise categorised as sporadic cases. A national outbreak criterion of at least three non-household cases was feasible to use in a very low incidence setting where similar HAV strains are seldom encountered unrelatedly. If used prospectively, it is also valuable to detect common clusters early, trigger an investigation and prevent larger outbreaks. Such low thresholds cannot be applied in high incidence settings with endemic circulation of highly similar or identical strains.

### Conclusions

This study confirmed a high impact of sequence-based typing for the detection of HAV outbreak cases and outbreaks in Sweden, and advocates for microbiological hepatitis A surveillance programs to include sequencing of HAV strains. Importantly, sequence analysis should be used to support outbreak investigations together with epidemiological data, as has been demonstrated in previous outbreaks in Sweden. In our experience, prompt sequencing, timely analysis, the maintenance of a national database and consulting the international database HAVNet strongly facilitates data interpretation. Sharing sequences in HAVNet, and by other means, internationally, ensures recognition of circulating strains and facilitates collaboration. While comprehensive sampling and typing would allow the most complete overview of HAV strains in a country, limited resources likely demand selection of strains to be sequenced, especially in a medium to high incidence setting. In such settings, studies evaluating the cost-effectiveness of sequence-based typing are needed in order to recommend such programmes from a societal perspective.

## References

[1] Enkirch T Eriksson R Persson S Schmid D Aberle SW Löf E Hepatitis A outbreak linked to imported frozen strawberries by sequencing, Sweden and Austria, June to September 2018. Euro Surveill. 2018;23(41). 10.2807/1560-7917.ES.2018.23.41.1800528 30326994 PMC6194910

[2] Gillesberg Lassen S Soborg B Midgley SE Steens A Vold L Stene-Johansen K Ongoing multi-strain food-borne hepatitis A outbreak with frozen berries as suspected vehicle: four Nordic countries affected, October 2012 to April 2013. Euro Surveill. 2013;18(17):20467. 23647625

[3] Nordic Outbreak Investigation Team. Joint analysis by the Nordic countries of a hepatitis A outbreak, October 2012 to June 2013: frozen strawberries suspected. Euro Surveill. 2013;18(27). 10.2807/1560-7917.ES2013.18.27.20520 23870076

[4] Ndumbi P Freidl GS Williams CJ Mårdh O Varela C Avellón A Hepatitis A outbreak disproportionately affecting men who have sex with men (MSM) in the European Union and European Economic Area, June 2016 to May 2017. Euro Surveill. 2018;23(33). 10.2807/1560-7917.ES.2018.23.33.1700641 30131095 PMC6205254

[5] Scavia G Alfonsi V Taffon S Escher M Bruni R Medici D A large prolonged outbreak of hepatitis A associated with consumption of frozen berries, Italy, 2013-14. J Med Microbiol. 2017;66(3):342-9. 10.1099/jmm.0.000433 28086079

[6] Fitzgerald M Thornton L O’Gorman J O’Connor L Garvey P Boland M Outbreak of hepatitis A infection associated with the consumption of frozen berries, Ireland, 2013--linked to an international outbreak. Euro Surveill. 2014;19(43):20942. 10.2807/1560-7917.ES2014.19.43.20942 25375902

[7] Severi E Verhoef L Thornton L Guzman-Herrador BR Faber M Sundqvist L Large and prolonged food-borne multistate hepatitis A outbreak in Europe associated with consumption of frozen berries, 2013 to 2014. Euro Surveill. 2015;20(29):21192. 10.2807/1560-7917.ES2015.20.29.21192 26227370

[8] Boxman IL Verhoef L Vennema H Ngui SL Friesema IH Whiteside C International linkage of two food-borne hepatitis A clusters through traceback of mussels, the Netherlands, 2012. Euro Surveill. 2016;21(3):30113. 10.2807/1560-7917.ES.2016.21.3.30113 26836217

[9] MacDonald E Steens A Stene-Johansen K Gillesberg Lassen S Midgley S Lawrence J Increase in hepatitis A in tourists from Denmark, England, Germany, the Netherlands, Norway and Sweden returning from Egypt, November 2012 to March 2013. Euro Surveill. 2013;18(17):20468. 23647624

[10] Gassowski M Michaelis K Wenzel JJ Faber M Figoni J Mouna L Two concurrent outbreaks of hepatitis A highlight the risk of infection for non-immune travellers to Morocco, January to June 2018. Euro Surveill. 2018;23(27). 10.2807/1560-7917.ES.2018.23.27.1800329 29991381 PMC6152161

[11] Tavoschi L Severi E Carrillo Santisteve P Lopalco P. Hepatitis A in the EU/EEA: The case for scaling up prevention. Vaccine. 2018;36(19):2501-3. 10.1016/j.vaccine.2018.02.100 29628152

[12] Lemon SM, Ott JJ, Van Damme P, Shouval D. Type A viral hepatitis: A summary and update on the molecular virology, epidemiology, pathogenesis and prevention. J Hepatol. 2017;S0168-8278(17)32278-X. 28887164 10.1016/j.jhep.2017.08.034

[13] Matheny SC Kingery JE Hepatitis A Hepatitis A. Hepatitis A. Am Fam Physician. 2012;86(11):1027-34, quiz 1010-2. 23198670

[14] Kroneman A de Sousa R Verhoef L Koopmans MPG Vennema H On Behalf Of The HAVNet Network. Usability of the international HAVNet hepatitis A virus database for geographical annotation, backtracing and outbreak detection. Euro Surveill. 2018;23(37). 10.2807/1560-7917.ES.2018.23.37.1700802 30229723 PMC6144472

[15] Nainan OV Xia G Vaughan G Margolis HS. Diagnosis of hepatitis a virus infection: a molecular approach. Clin Microbiol Rev. 2006;19(1):63-79. 10.1128/CMR.19.1.63-79.2006 16418523 PMC1360271

[16] Werber D Michaelis K Hausner M Sissolak D Wenzel J Bitzegeio J Ongoing outbreaks of hepatitis A among men who have sex with men (MSM), Berlin, November 2016 to January 2017 - linked to other German cities and European countries. Euro Surveill. 2017;22(5). 10.2807/1560-7917.ES.2017.22.5.30457 28183391 PMC5388120

[17] Bruni R Taffon S Equestre M Chionne P Madonna E Rizzo C Key Role of Sequencing to Trace Hepatitis A Viruses Circulating in Italy During a Large Multi-Country European Foodborne Outbreak in 2013. PLoS One. 2016;11(2):e0149642. 10.1371/journal.pone.0149642 26901877 PMC4764681

[18] Guzman-Herrador BR Panning M Stene-Johansen K Borgen K Einöder-Moreno M Huzly D Importance of molecular typing in confirmation of the source of a national hepatitis A virus outbreak in Norway and the detection of a related cluster in Germany. Arch Virol. 2015;160(11):2823-6. 10.1007/s00705-015-2531-y 26249822

[19] Enkirch T Severi E Vennema H Thornton L Dean J Borg ML Improving preparedness to respond to cross-border hepatitis A outbreaks in the European Union/European Economic Area: towards comparable sequencing of hepatitis A virus. Euro Surveill. 2019;24(28). 10.2807/1560-7917.ES.2019.24.28.1800397 31311618 PMC6636214

[20] National Institute for Public Health and the Environment (RIVM). HAVNet. Bilthoven: RIVM. [Accessed: 31 Oct 2019]. Available from: www.havnet.nl

[21] Statistics Sweden (SCB). Solna: SCB. [Accessed: 28 Oct 2019]. Available from: https://www.scb.se/hitta-statistik/statistik-efter-amne/befolkning/befolkningens-sammansattning/befolkningsstatistik/pong/tabell-och-diagram/helarsstatistik--riket/befolkningsstatistik-i-sammandrag/

[22] European Commission. Commission implementing decision (EU) 2018/945 of 22 June 2018 on the communicable diseases and related special health issues to be covered by epidemiological surveillance as well as relevant case definitions. Official Journal of the European Union. Luxembourg: Publications Office of the European Union. 6 Jul 2018. Available from: https://eur-lex.europa.eu/legal-content/EN/TXT/PDF/?uri=CELEX:32018D0945&from=EN#page=29

[23] Saitou N Nei M. The neighbor-joining method: a new method for reconstructing phylogenetic trees. Mol Biol Evol. 1987;4(4):406-25. 3447015 10.1093/oxfordjournals.molbev.a040454

[24] Tamura K Nei M. Estimation of the number of nucleotide substitutions in the control region of mitochondrial DNA in humans and chimpanzees. Mol Biol Evol. 1993;10(3):512-26. 8336541 10.1093/oxfordjournals.molbev.a040023

[25] European Centre for Disease Prevention and Control (ECDC). Hepatitis A virus in the EU/EEA, 1975-2014. Technical Report. Stockholm: ECDC; 2016. Available from: https://www.ecdc.europa.eu/en/publications-data/hepatitis-virus-eueea-1975-2014

[26] Carrillo-Santisteve P Tavoschi L Severi E Bonfigli S Edelstein M Byström E Seroprevalence and susceptibility to hepatitis A in the European Union and European Economic Area: a systematic review. Lancet Infect Dis. 2017;17(10):e306-19. 10.1016/S1473-3099(17)30392-4 28645862

[27] Gossner CM Severi E Danielsson N Hutin Y Coulombier D. Changing hepatitis A epidemiology in the European Union: new challenges and opportunities. Euro Surveill. 2015;20(16):21101. 10.2807/1560-7917.ES2015.20.16.21101 25953274

[28] Petrignani M Verhoef L Vennema H van Hunen R Baas D van Steenbergen JE Underdiagnosis of foodborne hepatitis A, The Netherlands, 2008-2010(1.). Emerg Infect Dis. 2014;20(4):596-602. 10.3201/eid2004.130753 24655539 PMC3966399

[29] Persson H Nasta F Svensson I Widerström M. Oväntat hög andel förskolebarn immuna mot hepatit A-virus - Smittspårningsutredning visar på behovet av vaccination inför utlandsresor. [Unexpectedly high proportion of preschool children immune to hepatitis A virus. Contact tracing investigation shows the need for vaccination prior to traveling abroad]. Swedish. Lakartidningen. 2014;111(49-50):2224-6. 25462320

